# Supplementary figures and images for: Systematic evaluation of the gut microbiome of swamp eel (Monopterus albus) by 16S rRNA gene sequencing
Source: PeerJ. 2019 Dec 20;7:e8176. doi: 10.7717/peerj.8176 (PMC6927349; doi:10.7717/peerj.8176)

Rarefaction Measure: Observed\_OTUs

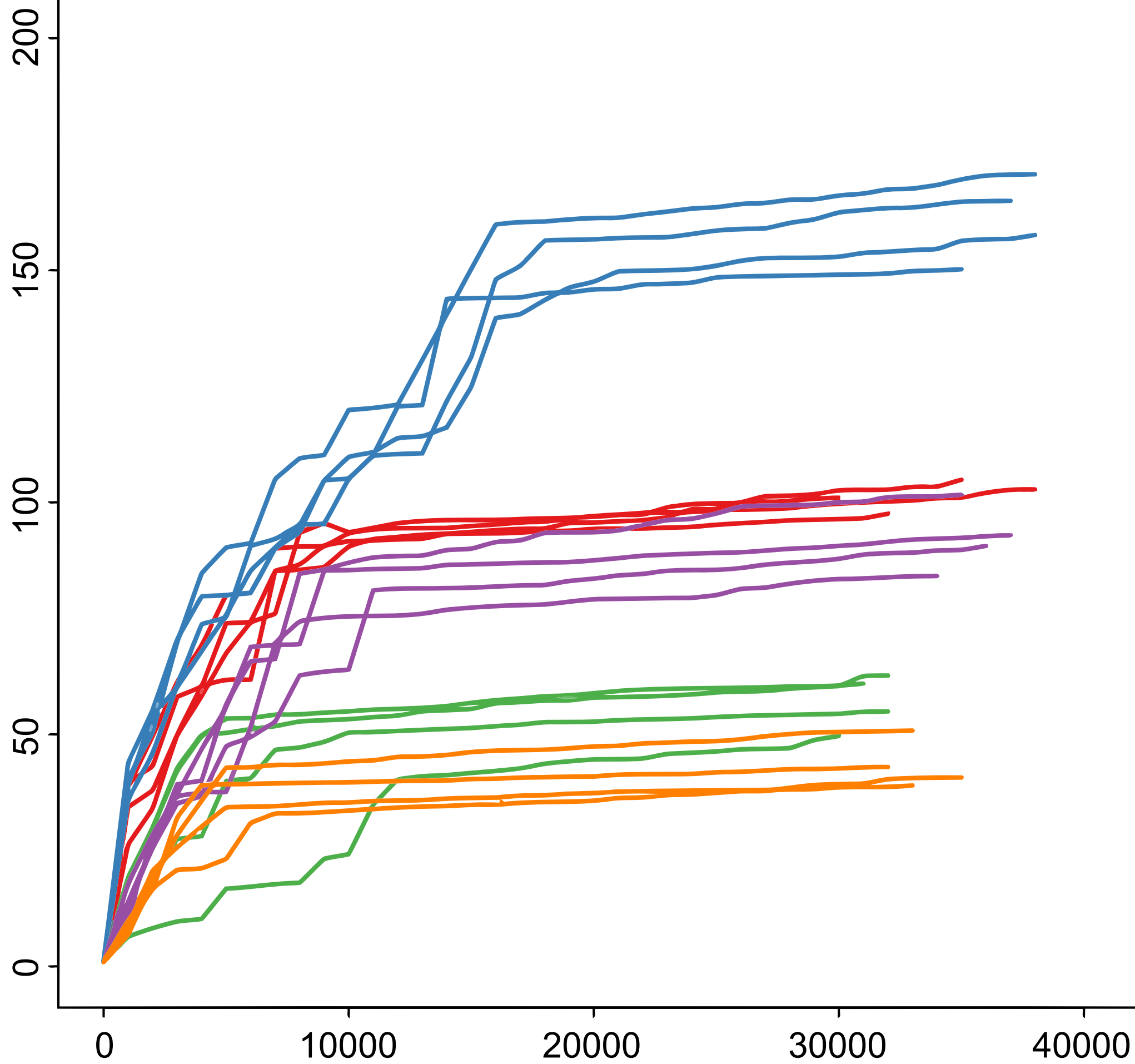

MC  
MM  
HC  
HM  
S

Nuber of sequences sampled

Supplement: Supplemental Information 1 [file peerj-07-8176-s001.pdf]

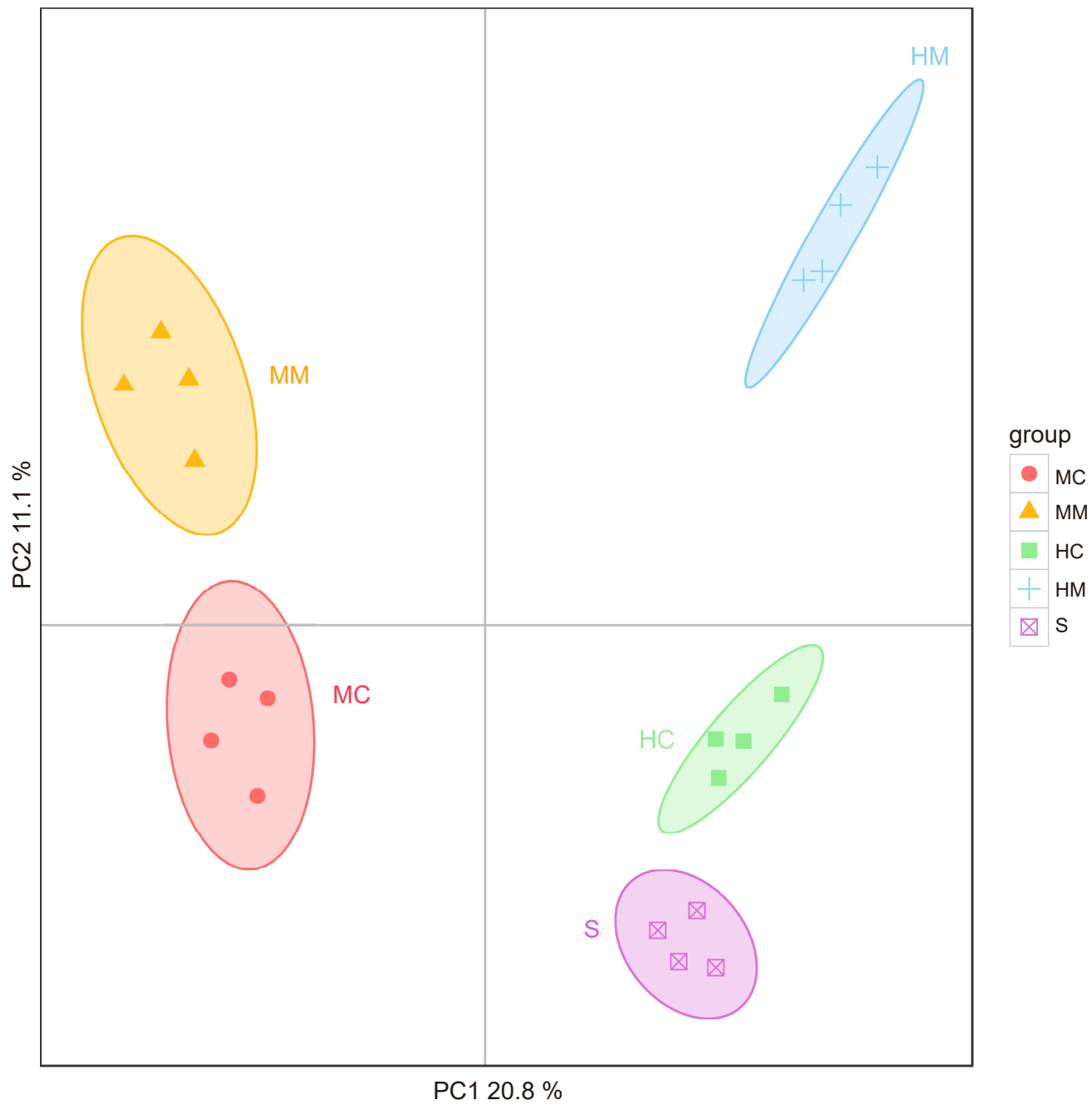

Supplement: Supplemental Information 2 — Principal Coordinate Analysis (PCoA) of microbialcommunity in different gut compartments based on the weighted UniFrac distancematrix (n = 4). The individual samples are color- and shape-coordinated according to the gut compartment. MC, midgut content; MM, midgut compartment; HC, hindgut content; HM, hindgut mucosa; S, stools. [file peerj-07-8176-s002.pdf]
